# Ex Situ Fabrication of Polypyrrole-Coated Core-Shell Nanoparticles for High-Performance Coin Cell Supercapacitor

**DOI:** 10.3390/nano8090726

**Published:** 2018-09-14

**Authors:** Hoseong Han, Sunghun Cho

**Affiliations:** School of Chemical Engineering, Yeungnam University, Gyeongsan 38541, Korea; byecome123@gmail.com

**Keywords:** coin cell supercapacitor, two electrode supercapacitor, polypyrrole, core-shell nanoparticle, solution process

## Abstract

Silica-conducting polymer (SiO_2_-CP) has the advantages of high electrical conductivity, structural stability, and the facile formation of thin-film. This work deals with the preparation and optimization of polypyrrole (PPy)-encapsulated silica nanoparticles (SiO_2_ NPs) using an ex situ method. The SiO_2_-PPy core-shell NPs prepared by the ex situ method are well dispersed in water and facilitate the mass production of thin-film electrodes with improved electrical and electrochemical performances using a simple solution process. As-prepared SiO_2_-PPy core-shell NPs with different particle sizes were applied to electrode materials for two-electrode supercapacitors based on coin cell batteries. It was confirmed that the areal capacitance (73.1 mF/cm^2^), volumetric capacitance (243.5 F/cm^3^), and cycling stability (88.9% after 5000 cycles) of the coin cell employing the ex situ core-shell was superior to that of the conventional core-shell (4.2 mF/cm^2^, 14.2 mF/cm^3^, and 82.2%). Considering these facts, the ex situ method provides a facile way to produce highly-conductive thin-film electrodes with enhanced electrical and electrochemical properties for the coin cell supercapacitor application.

## 1. Introduction

Conducting polymer (CP) is a class of organic materials that can generate electricity, and has attracted a great deal of attention due to its various advantages including high electrical conductivity, low cost, easy synthesis process, tunable doping level, and so forth [1,2]. After suitable doping processes, the electrons can move freely through the conjugated structure inside the CP chains so that the CP can conduct electric current. Polypyrrole (PPy) is a p-doped conducting polymer composed of repetitive bonds of five-membered heterocyclic rings including amine (N–H) groups, thus allowing the formation of hydrogen bonding forces between PPy chains [1,2]. Due to such structural characteristics, PPy can achieve thermal stability up to 150 °C. Therefore, various studies on the synthetic methods of PPy have been made through the chemical oxidation polymerization and the electrochemical polymerization [1,2,3,4,5,6,7,8,9,10,11,12].

Proper control of the shape of the PPy is very important for high performance in a variety of applications, such as supercapacitors, chemical sensors, solar cells (SCs), fuel cells (FCs) and so forth [3,4,5,6,7,8]. When PPy is made of nanomaterials, it has following advantages over bulk materials: (1) higher surface area; (2) better reactivity; (3) enhanced electrical conductivity [1,2,4,6,10,11,12,13,14,15,16,17,18,19,20]. Studies on the synthesis of nanomaterials with various shapes such as nanorods (NRs), nanoparticles (NPs), and nanotubes (NTs) have been carried out using template-mediated methodologies [1,2,3,4,5,6,7,8,9,10,11,12,13,14,15,16,17,18,19,20]. However, polymers including PPy are swollen and collapsed in the process of adsorption and desorption of electrolyte ions, resulting in problems of greatly reducing the performance and reliability of the electrode material [3,4,5,8]. In addition, CPs including PPy have relatively low surface area (10^0^–10^1^ m^2^ g^−1^), lowering the ion exchange between the electrode and electrolyte [1,19].

Among the various nanomaterials, the core-shell is very suitable as a stable electrode material because the Si core can protect the CP shell from swelling and shrinkage problems [8,9,10,11,12,13,14,15,16,17,18,19]. In addition, the high surface area of the Si cores enable rapid adsorption/desorption of electrolyte ions within the electrodes [19]. On the other hand, the CP shell plays roles in reinforcing the poor electrical properties of Si cores [13,18,19]. Thus, the synergistic effect from the PPy shell and the Si core would be advantageous for making a supercapacitor electrode that provides robustness and high electroactivity. Jang and co-workers have fabricated a variety of SiO_2_-CP core-cells, including PPy, polyaniline (PANI), and polythiophene (PT) [18,19]. Furthermore, the electro-rheological properties of the prepared core-shell materials have also been tested [17,18]. A typical synthesis of these SiO_2_-CP core-shell structures was performed according to the in situ method wherein the protonated pyrrole monomer is polymerized on an anionic SiO_2_ NP surface [9,18]. In conventional synthesis, the SiO_2_ templates limit the growth of polymer chains, resulting in undesirable α,α′-linkages inside the PPy chains. In addition, the low electrical properties of SiO_2_-PPy composites are highly related to the low efficiency of core-shell formation in the in situ syntheses [10,11,12,13,18]. Therefore, there is a need for optimization and development for producing SiO_2_-PPy core-shells with high efficiency of core-shell formation, which may result in the improved electrical and electrochemical performances.

Proper selection of the cell type has become an important issue to ensure the practical application of nanomaterial-based supercapacitors. Since the capacitance measured from the three electrode cell is twice as large as that of the two-electrode cell, the three electrode supercapacitor cannot be practical in real life [20]. Although the SiO_2_-PPy aerogel was demonstrated as a three-electrode supercapacitor, the two-electrode supercapacitor using the SiO_2_-PPy core-shell nanostructure has seldom been reported [11]. In particular, cell configuration in the form of a coin cell is one of the most used batteries in real life [21,22,23,24]. Therefore, the coin cell supercapacitor is more effective in evaluating the actual performance of an electrode material as compared with a conventional three-electrode supercapacitor. Hybrid materials composed of transition metals and carbon materials have been used as electrode materials for coin cell supercapacitors [21,22,23,24]. In order to promote the practical use of coin cell supercapacitors for modern electronic applications, it is necessary to find electrode materials with high areal and volumetric capacitances.

We describe herein the preparation of SiO_2_-PPy core-shell NPs for use as electrode materials in the coin cell supercapacitors using an ex situ method. By using the ex situ method, the PPy shell can be produced without the influence of SiO_2_ NPs, and the resultant PPy will have higher doping level and better electrical performance than the conventional ones. Successful formation of the core-shell NPs can also be achieved through electrostatic interaction between the PPy chain and the SiO_2_ core surface [9,18,19]. The purpose of this study was to identify the optimal synthetic condition of SiO_2_-PPy core-shell for the coin cell supercapacitors. The ex situ method using the optimal size of SiO_2_ core was beneficial for the fabrication of coin cell supercapacitors, which showed superior electrical and electrochemical performances compared with the in situ method.

## 2. Materials and Methods

### 2.1. Materials

Pyrrole (99%), iron (iii) chloride hexahydrate (FeCl_3_·6H_2_O, 97%), and SiO_2_ NPs (12 and 22 nm) were purchased from Sigma-Aldrich (St. Louis, MO, USA). Brunauer–Emmett–Teller (BET) surface areas for 12 and 22 nm SiO_2_ NPs were 220 and 140 m^2^ g^−1^, respectively. Components for coin cell assembly including spring, space, negative case, and positive case were obtained from Wellcos Corporation (Gunpo, Korea). The polypropylene–polyethylene–polypropylene (PP–PE–PP) trilayer (thickness: 25 µm) was acquired from Celgard (Celgard 2400, Celgard, LLC., Charlotte, NC, USA). Coin cell components (CR2032 type), and nickel (Ni) foil (thickness: 0.03 mm) were obtained from MTI Corporation (MF-NiFoil-25u, MTI Corporation, Richmond, CA, USA).

### 2.2. Fabrication of SiO_2_-PPy Core-Shell Nanoparticles (NPs)

0.56 g of FeCl_3_·6H_2_O was dissolved in 80 mL of deionized water (DI, Daejung Chemical & Metal, Co., Ltd., Siheung, Korea) followed by vigorous stirring at room temperature for an hour. The FeCl_3_ solution was mixed with 0.30 g of pyrrole monomers, and stirred vigorously at 3 °C for an hour to polymerize PPy. Dark and brownish precipitates of PPy were obtained by centrifugation of the prepared PPy solution. Dispersion of the SiO_2_ NPs was conducted by an hour of mechanical stirring at a stirring speed of 400 rpm and sonication treatment for an hour. The PPy precipitates become highly dispersible with 30 mL of SiO_2_ solution (0.8 wt.% with respect to distilled water) after 3 h of vigorous mechanical stirring at a stirring speed of 400 rpm and an hour of sonication treatment. The sonication treatments of the samples were conducted out using an ultrasonic bath (CPX2800H-E, Branson Ultrasonics Co., Danbury, CT, USA) with 110 W power and 40 kHz frequency. Morphological images of the SiO_2_-PPy core-shell NPs were recorded on a transmission electron microscope (TEM, LIBRA 120, Carl Zeiss, Oberkochen, Germany) and a field emission scanning electron microscope (FE-SEM, S-4800, HITACHI, LTD, Hitachi, Japan). To investigate the chemical compositions and doping states of the samples, X-ray photoelectron spectroscopy (XPS) spectra were recorded on a K-Alpha XPS instrument (Thermo K-Alpha XPS, Thermo Fisher Scientific, Waltham, MA, USA). X-ray diffractograms (XRDs) were measured with a X-ray diffractometer (X’Pert PRO, PANalytical, Westborough, MA, USA). Electrical conductivity of the core-shell NPs was measured using a 4-point probe conductivity meter (Mode Systems Co., Hanam, Korea) equipped with a current source meter (Keithley 2400, Keithley Co., Cleveland, OH, USA).

### 2.3. Assembly of Coin Cells Employing SiO_2_-PPy Core-Shell NPs

0.3 mL of SiO_2_-PPy core-shell NPs (6 mg mL^−1^ in distilled water) were deposited on current collectors made of Ni foil, and the samples were dried at 25 °C using a vacuum oven. The thickness of the SiO_2_-PPy films was about 3 µm, and the films were cut into circular shapes having a diameter of 15 mm. The PP–PE–PP trilayer film was cut into a circular-shaped membrane having a diameter of 17 mm. The resulting films and membrane were immersed in 1 M KOH solution for 3 h. The core-shell electrodes and the PP–PE–PP membrane were combined for the fabrication of coin cells, which were sealed using a hydraulic crimping machine (MSK-110, MTI Corporation, Richmond, CA, USA). 

### 2.4. Electrochemical Measurements

Evaluation of the electrochemical characteristics on the coin cells employing the SiO_2_-PPy core-shell NPs were carried out using a ZIVE SP2 electrochemical workstation (Wonatech, Seoul, Korea). Cyclic voltammetry (CV) and galvanostatic charge/discharge measurements were conducted in 1 M KOH electrolyte solution. CVs of the samples were measured from 0 and 1.0 V at scan rates 10 from 100 mV s^−1^. Galvanostatic charge/discharge experiments were performed by cycling the potential from 0 to 1.0 V at a current of 1 mA/cm^2^. Areal specific capacitances (C_A_’s) of the coin cells were calculated using the equation C_A_ (mF/cm^2^) = ∫IΔv/vAΔV [21,25,26,27,28]. Volumetric specific capacitances (C_V_’s) of the coin cells were calculated using the equation C_V_ (mF/cm^3^) = ∫IΔv/vLΔV [21,25,26,27,28]. Mass specific capacitances (C_m_’s) of the coin cells were calculated using the equation C_m_ (F/g) = ∫IΔv/mΔV [21,25,26,27,28]. In the equations of C_A_, C_V_, and C_m_, the terms ∫I, v, ΔV, A, L, and m indicate the integrated area under the CV, scan rate, potential window, electrode area, electrode volume, and electrode mass, respectively. Energy density of the coin cell was calculated according to the equation E (Wh/cm^3^) = C_V_ΔV^2^/2, where C_V_ and ΔV indicate the volumetric capacitance of each coin cell and voltage drop upon discharge, respectively [21,25,26,27,28]. Power density of the coin cell was calculated using the equation P (W/cm^3^) = E/t, where E and t indicate the energy density and discharging time of each coin cell, respectively [19,24,25,26,27]. Electrochemical impedance spectra (EIS) of the electrochemical cells were observed in the frequency range of 1 MHz to 10 mHz.

## 3. Results and Discussion

**Figure 1:**
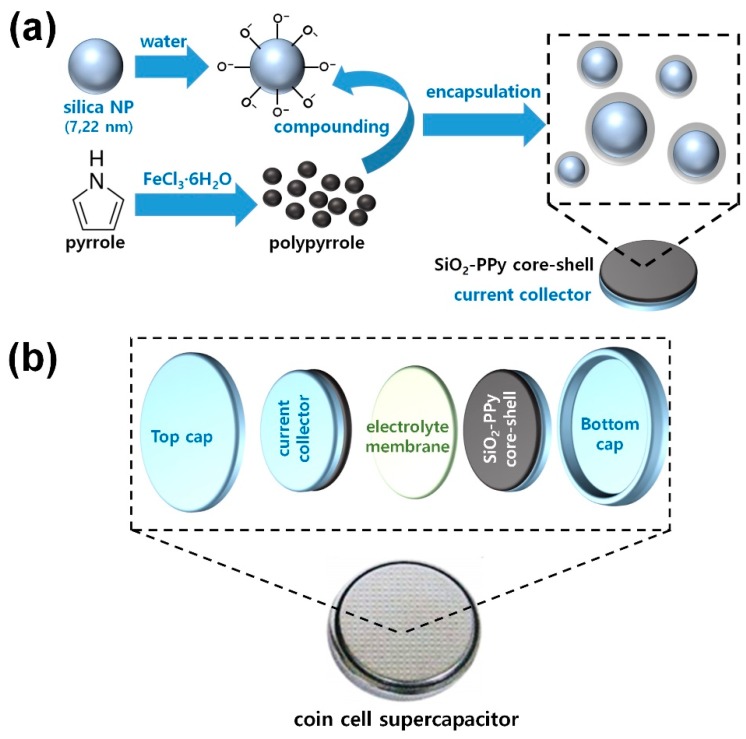
(**a**) Overall procedures for fabricating the SiO_2_-PPy core-shell nanoparticles (NPs) via ex situ method; (**b**) a structure of coin cell used in this research.

Overall procedures for fabricating the SiO_2_-PPy core-shell NPs are demonstrated in Figure 1a. In the ex situ method, polymerization of PPy and oxidation of SiO_2_ NPs were carried out respectively. During the dispersion process of SiO_2_ NPs within the distilled water, the surface of SiO_2_ NPs became negatively charged [1,18,19]. FeCl_3_·6H_2_O was acted as both a dopant and an oxidant for chemical oxidation polymerization of pyrrole monomers [9]. In this ex situ system, the PPy chains can be formed properly without any interference from the SiO_2_ NPs, which may result in fewer structural defects of PPy chains. As-prepared PPy precipitates were introduced into the SiO_2_ solution, and then the SiO_2_ NPs could be encapsulated by PPy shells through the mechanical and sonication treatments. Molecular interactions between the negatively charged SiO_2_ surfaces and the protonated PPy chains enable a successful formation of the core-shell structure [1,2,9,18,19]. The SiO_2_ core imparts structural stability to the core-shell structure to support the PPy shell. A dispersion solution of the core-shell NPs was fabricated into a thin-film electrode on the current collector to be used as electrodes in a coin cell. Successful film formation of the core-shell NPs was possible through a simple drop-casting process without using steric stabilizers. Figure 1b describes a general structure of the coin cell used in this research. The coin cell was assembled as a symmetrical supercapacitor, and the coin cell is composed of two core-shell electrodes, a PP–PE–PP electrolyte separator, a top cap, and a button cap. Ni foils were used as current collectors to ensure good electrical contact between the core-shell electrodes and the outer electrodes. A PP–PE–PP membrane was placed between the core-shell coated electrodes, and the membrane provided a porous path for the electrolyte ions. The electrolyte used in this work was 1 M KOH aqueous solution.

**Figure 2:**
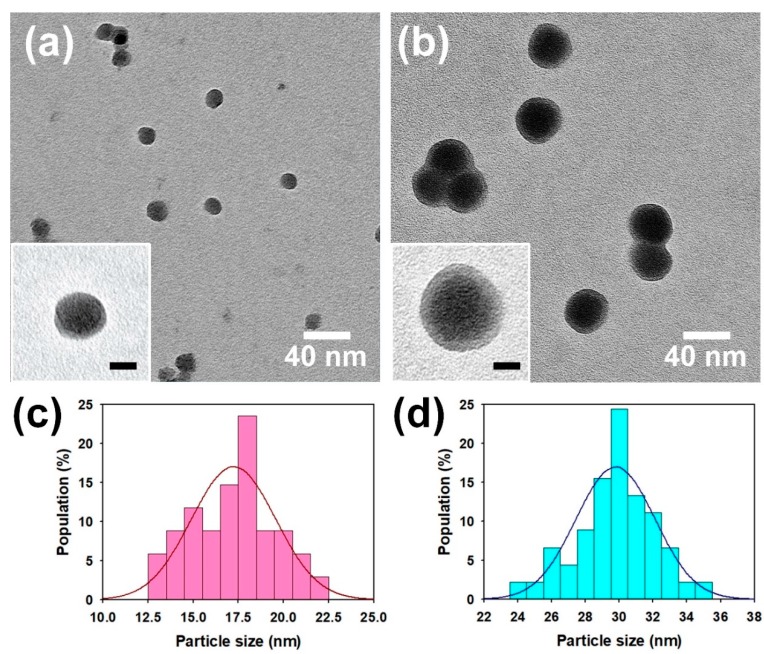
Transmission electron microscope (TEM) images of SiO_2_-PPy core-shell NPs with (**a**) 12 nm and (**b**) 22 nm SiO_2_ NPs prepared by the ex situ method. Histograms for particle size distribution for SiO_2_-PPy core-shell NPs with (**c**) 12 nm and (**d**) 22 nm SiO_2_ NPs prepared by ex situ method. The scale bars of inset images correspond to 10 nm. The number of core-shell NPs analyzed were 100 each.

Figure 2a,b represent the TEM images of the SiO_2_-PPy core-shell NPs prepared in different sizes of cores (12 nm and 22 nm). The results confirm that the size of the core-shell NPs could be controlled by changing the size of core NPs, and the core-shell NPs were well dispersed. The insets in Figure 2 clearly prove the successful formation of the SiO_2_ NPs by PPy shells. The shell thicknesses of the samples employing 12 nm SiO_2_ and 22 nm SiO_2_ cores were 2.5 ± 0.6 and 4 ± 1 nm, respectively. The average diameter and diameter distribution of the core-shell NPs were clarified by histograms of the particle size distributions (Figure 2c,d). The average sizes of core-shell NPs prepared with 12 nm and 22 nm SiO_2_ cores were ca. 17.2 ± 2.2 and ca. 29.3 ± 2.3 nm, respectively. The sizes of the NPs shown in the TEM images corresponded to the FE-SEM images (Figure S1, see Supplementary Materials). The presence of PPy in the SiO_2_-PPy core-shell was confirmed by comparing the element compositions of SiO_2_ and SiO_2_-PPy core-shell using scanning electron microscope-energy-dispersive X-ray (SEM-EDAX) mode (Table S1, see Supplementary Materials). From these results, it is considered that the core-shell was successfully synthesized via the ex situ method and that the diameter of the core-shell NP could be controlled by varying the size of the SiO_2_ core.

**Figure 3:**
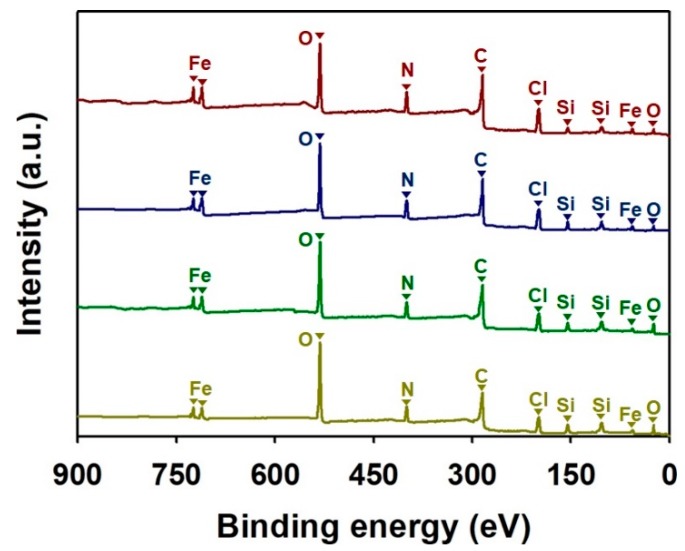
Fully scanned X-ray photoelectron spectroscopy (XPS) spectra of ex situ 12 nm (red), ex situ 22 nm (blue), in situ 12 nm (green), and in situ 22 nm (olive green).

**Figure 4:**
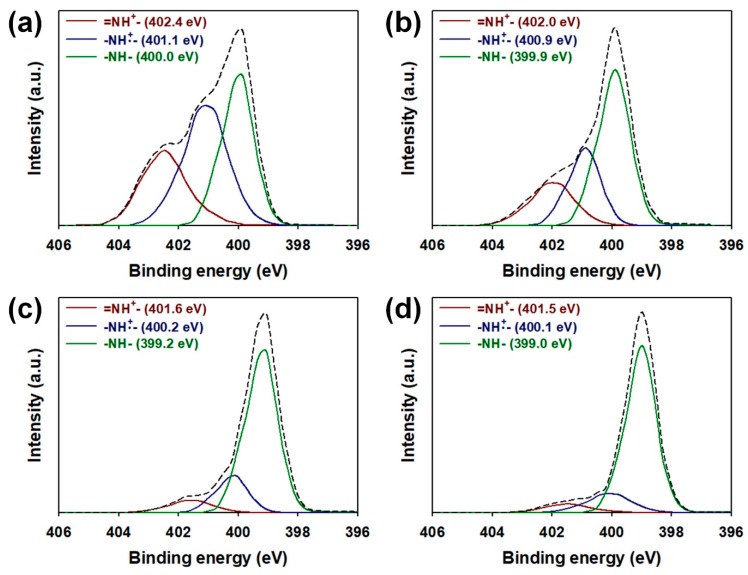
XPS core spectra in the N(1s) region of (**a**) ex situ 12 nm; (**b**) ex situ 22 nm; (**c**) in situ 12 nm, and (**d**) in situ 22 nm.

XPS was used to investigate changes in the elemental compositions and doping states of the SiO_2_-PPy core-shell NPs (Figure 3 and Figure 4). Figure 3 shows the fully scanned XPS patterns of the core-shell NPs. Every sample exhibited distinctive peaks at 284, 399, 531, 24, 103, 155, 711, 56, and 198 eV corresponding to C(1s), N(1s), O(1s), O(2s), Si(2p), Si(2s), Fe(2p), Fe(3p), and Cl(2p), respectively [10,29,30]. The samples obtained from the ex situ method (ex situ 12 nm and ex situ 22 nm) showed higher intensities at the peaks for C(1s), N(1s), Fe(2p), Fe(3p), and Cl(2p), while the samples obtained from the in situ method (in situ 12 nm and in situ 22 nm) exhibited higher Si content (Table S2, see Supplementary Materials). The peaks for C(1s), N(1s), Fe(2p), Fe(3p), and Cl(2p) are attributed to the PPy doped by FeCl_3_, and the peaks for Si(2p) and Si(2s) are originated from the SiO_2_ core. Therefore, it is considered that the ex situ method is more effective for forming the PPy shell on the Si surface than the in situ method.

Figure 4 represents the N(1s) core spectra of the core-shell NPs prepared by ex situ and in situ methods. The core-shell NPs showed three peaks at 399–400, 400.1–401.1, and 401.5–402.4 eV, corresponding to –NH– (neutral amine nitrogen), –NH•^+^ (polaron), and =NH^+^ (bipolaron), respectively. To evaluate the doping states of samples, the ratio of N^+^ species (sum of –NH•^+^ and =NH^+^) to N species (sum of –NH–, –NH•^+^ and =NH^+^) was calculated [29,30]. This ratio was 0.57, 0.44, 0.23 and 0.14 for the ex situ 12 nm, ex situ 22 nm, in situ 12 nm, and in situ 22 nm, respectively (Table S3, see Supplementary Materials). The results prove that the formation of PPy shells in the ex situ method is less affected by SiO_2_ NP, resulting in less structural defects and more charge carriers than the in situ method. The enhanced doping level of the ex situ sample enables extended conduction paths for delocalizing more electrons, resulting in improved conductivity of the core-shell structure. In addition, it was found that the core-shell NPs with 12 nm SiO_2_ core has higher levels of protonation than the sample using 22 nm SiO_2_ core. This implies that the core-shell NP with the smaller size is advantageous in forming more compact and continuous conduction pathways than the larger one. In the XRD patterns of samples, every sample only exhibited a broad characteristic band of PPy at 2Ɵ = 26°, while the characteristic peak of SiO_2_ could not be distinguished [30]. This suggests that the SiO_2_ NPs were highly intercalated into the PPy shells (Figure S2, see Supplementary Materials).

**Figure 5:**
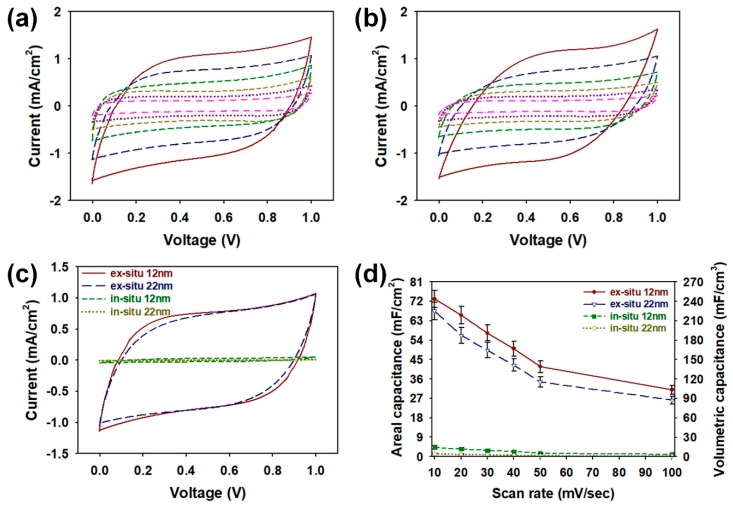
Cyclic voltammetry (CV) curves of coin cells containing core-shell NPs with (**a**) 12 nm and (**b**) 22 nm SiO_2_ NPs prepared by ex situ method at different scan rates: 100 mV/s (red), 50 mV/s (blue), 40 mV/s (green), 30 mV/s (olive green), 20 mV/s (purple), and 10 mV/s (pink); (**c**) CV curves of coin cells containing core-shell NPs prepared by different synthetic conditions at a scan rate of 50 mV/s; (**d**) plots of areal capacitance (mF/cm^2^) and volumetric capacitance (mF/cm^3^) for coin cells containing core-shell NPs prepared by different synthetic conditions with increasing scan rates.

After morphological characterizations of the core-shell NPs, electrochemical evaluations of coin cells as shown in Figure 5 and Figure 7. The CV curves of the core-shell NPs were measured in a 1 M KOH electrolyte at scan rates from 10 to 100 mV/s (Figure 5a,b). Every CV curve appeared as typical rectangular responses, and these rectangular-shaped CV curves indicate that the SiO_2_-PPy core-shell are suitable for a coin cell supercapacitor [21,25]. Among the samples prepared by the ex situ method, the sample employing 12 nm SiO_2_ core has shown a slightly larger CV area than that of the sample with 22 nm SiO_2_ core at every scan rate. As the size of core-shell NP decreases, the surface areas of core-shell structures for interacting with electrolyte ions increases [19]. Therefore, the coin cell employing the ex situ 12 nm can store more electric charges compared with the ex situ 22 nm sample. In comparison with the CV curves of samples prepared by the in situ method (in situ 12 nm and in situ 22 nm), larger CV areas were observed for samples obtained by the ex situ method (ex situ 12 nm and ex situ 22 nm) (Figure 5c). The results support the hypothesis that PPy polymerized by the ex situ method will have fewer structural defects and thus will achieve higher electrochemical performance. To achieve miniaturation of supercapacitors for state-of-art electronic applications, it is crucial to ensure high areal capacitance (C_A_) and volumetric capacitance (C_V_). Therefore, the C_A_ and C_V_ of the samples were evaluated at different scan rates (Figure 5d). As the scan speed increases, electrolyte ion diffusion inside the core-shell structure becomes more difficult, thereby reducing both the C_A_ and C_V_ of the samples [21]. The maximum C_A_ (mF/cm^2^) of ex situ 12 nm cell was about 73.1, which was higher than that of ex situ 22 nm (67.5), in situ 12 nm (4.2), and in situ 22 nm (1.3). The C_V_ (mF/cm^3^) increased in the following order: in situ 22 nm (4.4) < in situ 12 nm (14.2) < ex situ 22 nm (225.1) < ex situ 12 nm (243.5). Same tendency was also observed for the mass capacitance (C_m_, F/g) values at a scan rate of 10 mV/s in the following order: in situ 22 nm (1.4) < in situ 12 nm (4.2) < ex situ 22 nm (66.8) < ex situ 12 nm (72.4). From these results, it was evident that the ex situ method is more appropriate for constructing the high-performance core-shell NPs than that of conventional in situ method. Moreover, the smaller size of ex situ 12 nm offer larger electrode/electrolyte interface areas, which results in the enhanced accessibility through the electrolyte at the interface of PPy. Therefore, ex situ 12 nm with a larger surface area can exhibit the highest capacitive performance among the assembled coin cells [19].

**Figure 6:**
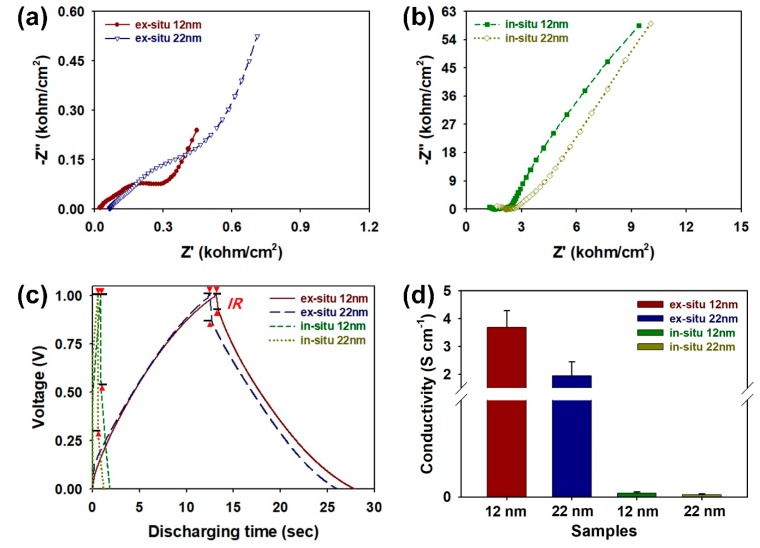
Nyquist impedance plots of coin cells containing core-shell NPs prepared by (**a**) ex situ and (**b**) in situ methods in the frequency range from 1 MHz to 10 mHz; (**c**) galvanostatic charge–discharge (GCD) curves of coin cells containing core-shell NPs prepared by different synthetic conditions at a current of 1 mA/cm^2^; (**d**) electrical conductivity of core-shell NPs prepared by ex situ and in situ methods.

To further identify the factors affecting the electrochemical performances of the core-shell NPs, Nyquist plots were measured using electrochemical impedance spectroscopy (EIS) analyses (Figure 6a,b). In the EIS plots for every sample, vertical straight lines were observed in the low frequency region. The results indicate the effective ion diffusions and appropriate capacitive behaviors for the core-shell NPs [23,25]. The equivalent series resistance (ESR) within the coin cells increased as following orders: ex situ 12 nm (2.26 × 10^1^ Ω/cm^2^) < ex situ 22 nm (6.43 × 10^1^ Ω/cm^2^) < in situ 12 nm (1.24 × 10^3^ Ω/cm^2^) < in situ 22 nm (1.64 × 10^3^ Ω/cm^2^). This suggests that the ex situ 12 nm sample has higher conductivity for the electrolyte and lower internal resistance compared to the other samples [23,25]. From the Nyquist plots, we could find out the effect of ex situ method and proper NP size on the electrochemical performance of coin cell. To evaluate the capacitive performances of coin cell supercapacitors employing the core-shell NPs with different synthetic conditions, galvanostatic charge–discharge (GCD) curves were acquired at a current of 1 mA/cm^2^ with a voltage from 0 to 1.0 V (Figure 6c). To achieve equilibration of charging and discharging currents, the GCD curves were measured after performing 20 CV cycles (Figure S3, see Supplementary Materials) [31]. Before 20 cycles of CV, imbalances between charging and discharging currents were observed in the GCD curve of each sample. This imbalance lowers the Coulombic efficiency of the sample. After 20 cycles of CV, it was found that the imbalances were resolved. The symmetrical shape of the charge–discharge curve is indicative of the reversible redox reactions of SiO_2_-PPy core-shell structures [23]. The internal resistance (IR) of the core-shell structures was estimated from the voltage drop at the onset of the discharge curves. The IRs within the coin cells increased by the following orders: ex situ 12 nm (3.07 × 10^1^ Ω/cm^2^) < ex situ 22 nm (5.67 × 10^1^ Ω/cm^2^) < in situ 12 nm (1.91 × 10^2^ Ω/cm^2^) < in situ 22 nm (2.89 × 10^2^ Ω/cm^2^). The IRs observed in the discharge curves were consistent with the EIS results. The results prove that the PPy shells of the ex situ samples could be polymerized without influences of the SiO_2_ cores, which result in smaller voltage drops in comparison to the conventional core-shell NPs prepared by the in situ method. The reduced voltage drops and IRs are highly related to enhanced conductivity of the ex situ samples. Electrical conductivity of the ex situ 12 nm, ex situ 22 nm, in situ 12 nm, and in situ 22 nm are shown in Figure 6d. Conductivity was calculated using the 4-point probe method, as described in the equation σ (S cm^−1^) = 1/*ρ* = (ln2)/(πt)1/R, where *ρ*, R, and t refer to the static resistivity, sheet resistivity, and thickness of the sample, respectively [12,13,19]. The conductivity of core-shell nanostructures increased as follows: in situ 22 nm (0.012 ± 0.005 S cm^−1^) < in situ 12 nm (0.02 ± 0.006 S cm^−1^) < ex situ 22 nm (1.94 ± 0.5 S cm^−1^) < in situ 12 nm (3.68 ± 0.6 S cm^−1^), which is consistent with the XPS, C_V_, C_A_, C_V_, EIS, and GCD results. The results indicate that the samples prepared by the ex situ method have higher levels of protonation due to the better production of the charge carriers within the PPy structure, as evidenced in Figure 3 and Figure 4. The core-shell NPs containing 12 nm SiO_2_ core have shown higher conductivity than that of the core-shell NPs with 22 nm SiO_2_ core. This proves that the conduction losses of the PPy shell can be lowered by reducing the size of the SiO_2_ core. The values obtained by our work have shown more than 10^6^–10^7^ times higher conductivity compared to the previous work on SiO_2_-CP composites, resulting in the improved ability to collect electric charges within the electrode [12,13]. Judging from these results, it was evident that the ex-method was effective to enhance the electrical and electrochemical properties of the SiO_2_-PPy core-shell NPs.

**Figure 7:**
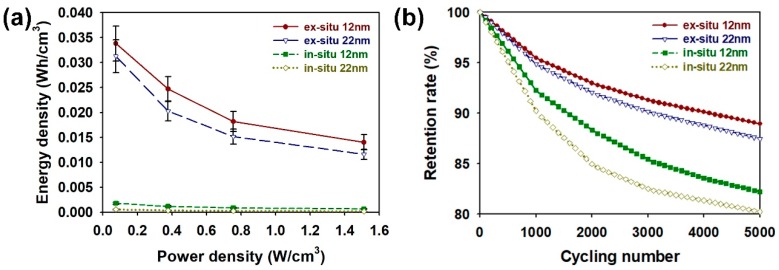
(**a**) Ragone plots and (**b**) cycling stability of coin cells containing core-shell NPs prepared by different synthetic conditions.

To examine the practical applicability of the ex situ 12 nm and 22 nm samples, Ragone plots and cycling stabilities of the coin cells employing ex situ 12 nm, ex situ 22 nm, in situ 12 nm, and in situ 22 nm are shown in Figure 7. The maximum energy density of ex situ 12 nm was 0.0338 Wh/cm^3^ with a power density of 0.0756 W/cm^3^, and decreased to 0.0140 Wh/cm^3^ with a power density of 0.151 W/cm^3^. It was found that the ex situ 12 nm sample showed higher energy density than the ex situ 22 nm. By comparison, the ex situ 12 nm store more energy compared to the ex situ 22 nm sample, indicating that the smaller particle size of ex situ 12 nm provides larger electrode surfaces for interacting with the electrolyte ions. The cycling stabilities of the coin cells based on the ex situ 12 nm, ex situ 22 nm, in situ 12 nm, and in situ 22 nm samples were measured with GCD cycles at a current density of 1 mA/cm^2^ (Figure 7b). After 5000 cycles, the retention rate of the core-shell NPs (given in %) increased in the following order: in situ 22 nm (80.2) < in situ 12 nm (82.2) < ex situ 22 nm (87.5) < in situ 12 nm (88.9). The samples obtained from the in situ method showed lower retention rates, suggesting that the in situ 12 nm and 22 nm samples suffer from low electrical conductivity due to the low efficiency of core-shell formation. The higher retention rates of ex situ samples imply that the PPy shell protects the SiO_2_ core from the KOH, which is a strong base that slowly dissolves the SiO_2_ NP [32]. On the other hand, these results proved that the core-shell NP with high surface area and good mechanical stability prevented the conductive PPy shell from swelling and collapsing upon repeated cycling [13,18,19]. Although the capacitance losses with increasing cycles were inevitable, the ex situ method based on the coin cell configuration was effective to magnify the synergistic effects from the PPy shell and SiO_2_ core, lowering the capacitance losses [21,22,23,24].

The overall performances of state-of-art two-electrode supercapacitors and our work are summarized in Table 1 [8,21,23,26,27,28]. Our work has shown higher or comparable capacitive characteristics in comparison to the previous work on two-electrode supercapacitors, indicating that the core-shell NPs prepared by the ex situ method is effective for realizing a high-performance coin cell supercapacitor.

## 4. Conclusions

SiO_2_-PPy core-shell NPs with an improved electrochemical performance were prepared by the simple ex situ method. The ex situ method ensured enhanced capacitive performances of the core-shell NPs and successful formation of the SiO_2_ NP by the PPy shell. The core-shell NPs prepared by the ex situ method exhibited lower internal resistance and improved capacitive behaviors compared with the in situ method. Furthermore, the optimal size of the SiO_2_ core was identified as 12 nm from the electrochemical characterizations, and the maximum areal capacitance, volumetric capacitance, and cycling stability reached up to 71.3 mF/cm^2^, 237.6 F/cm^3^, and 88.9% (after 5000 cycles), respectively. Significant improvements in the performance of the SiO_2_-PPy core-shell NP prepared by the ex situ method were highly related to the improved electrical conductivity over the samples obtained by the in situ method. Given that the miniaturation of supercapacitors with high capacitance per area and per volume is highly desired in real-world applications, the SiO_2_-PPy core-shell can be a promising candidate for high-performance coin cell supercapacitors.

## Figures and Tables

**Table 1:** Electrochemical performance of supercapacitors based on the two electrode cell.

Electrode Material	Voltage Window	Electrolyte	Specific Capacitance		Reference
			C_A_ (mF/cm^2^)	C_V_ (mF/cm^3^)	
CNT/PPy/MnO_2_	0.9 V	KCl	-	16.1	[8]
MoS_2_–graphene	1.0 V	1.0 M Na_2_SO_4_	11	-	[21]
MnO_2_/CNT-VN	1.8 V	0.5 M Na_2_SO_4_	-	43	[23]
MoS_2_ nanosheet	0.5 V	-	8	178	[26]
3D graphene-CNT	1.0 V	1.0 M Na_2_SO_4_	2.16	1.08	[27]
Graphene-CNT	1.0 V	1.0 M Na_2_SO_4_	-	130	[28]
SiO_2_-PPy core-shell	1.0 V	1 M KOH	71.3	237.6	This work

## Supplementary Materials

The following are available online at http://www.mdpi.com/2079-4991/8/9/726/s1, Figure S1: FE-SEM images of SiO_2_-PPy core-shell NPs with (a) 12 nm and (b) 22 nm SiO_2_ NPs prepared by ex situ method, Figure S2: XRD patterns of ex situ 12 nm (red), ex situ 22 nm (blue), in situ 12 nm (green), and in situ 22 nm (olive green), Figure S3: GCD curves of coin cells containing core-shell NPs before and after performing 20 CV cycles at a current of 1 mA/cm^2^: (a) ex situ 12 nm, (b) ex situ 22 nm, (c) in situ 12 nm, and (d) in situ 22 nm, Table S1: Elemental composition of pristine SiO_2_ and SiO_2_-PPy core-shell NP with 12 nm SiO_2_ core prepared by ex situ method, Table S2: Elemental composition of SiO_2_-PPy core-shell NPs obtained using XPS analyses, Table S3: Peak analyses of XPS core spectra in the N(1s) region of SiO_2_-PPy core-shell NPs.
