# Supplementary material for: Ex Situ Fabrication of Polypyrrole-Coated Core-Shell Nanoparticles for High-Performance Coin Cell Supercapacitor
Source: Nanomaterials (Basel). 2018 Sep 14;8(9):726. doi: 10.3390/nano8090726 (PMC6164064; doi:10.3390/nano8090726)
Supplement: Supplementary file 1 [file nanomaterials-08-00726-s001.pdf]

Supplementary Materials

Ex-situ Fabrication of Polypyrrole-coated Core-Shell Nanoparticles for High-Performance Coin Cell Supercapacitor

Hoseong Han and Sunghun Cho *

School of Chemical Engineering, Yeungnam University, Gyeongsan 38541, Korea; byecome123@gmail.com

* Correspondence: shcho83@ynu.ac.kr; Tel.: +82-53-810-2535

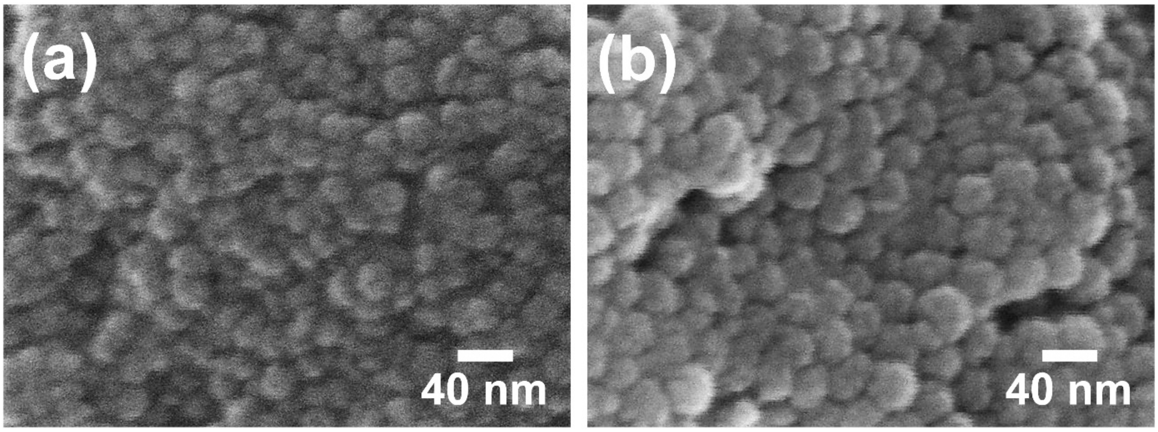

Figure S1. FE-SEM images of SiO_2-PPy core-shell NPs with (**a**) 12 nm and (**b**) 22 nm SiO_2 NPs prepared by ex-situ method.

Table S1. Elemental composition of pristine SiO_2 and SiO_2-PPy core-shell NP with 12 nm SiO_2 core prepared by ex-situ method.

Sample	Atomic ratio (%)[1]						C/N Value
	C	N	O	Si	Fe	Cl	
pristine SiO_2	5.79	-	53.66	40.55	-	-	-
SiO_2-PPy core-shell	24.02	7.37	42.70	10.76	12.98	2.16	3.26

[1]These values were obtained in the SEM-EDAX mode for 50 sec, at a beam current of 0.1 A. and an accelerating voltage of 10 kV.

The elemental compositions of the pristine SiO_2 NP and core-shell NP were obtained by using the energy-dispersive X-ray (EDAX) analysis data obtained from the FE-SEM instrument (**Table S1**). The %C value of core-shell was increased compared to the pristine SiO_2 NP, while the %O and %Si values decreased due to the presence of PPy shells on the SiO_2 surface. The detection of N is attributed to the amine group of PPy, thereby the existence of PPy shell on the surface of SiO_2 NP can be confirmed [18,19]. The discovery of Fe and Cl atoms in the core-shell also indicates that PPy shell is doped by

$FeCl_3 \cdot 6H_2O$. Observation of C atrom in the pristine SiO_2 sample can be attributed to the carbon tape used to hold the sample on the EDAX specimen mount [19].

Table S2. Elemental composition of SiO_2-PPy core-shell NPs obtained using XPS analyses.

Sample	Atomic ratio (%)[1]						C/N Value
	C	N	O	Si	Fe	Cl	
ex-situ 12nm	23.09	7.65	43.52	6.53	11.75	7.46	3.02
ex-situ 22nm	22.81	7.33	45.33	7.08	10.26	7.19	3.11
ex-situ 12nm	20.35	6.21	48.69	8.31	9.82	6.62	3.28
ex-situ 22nm	19.68	5.76	49.86	9.13	9.58	5.99	3.42

Table S3. Peak analyses of XPS core spectra in the N(1s) region of SiO_2-PPy core-shell NPs.

Samples	XPS Atomic Ratio			
	–NH–	$–NH^{\bullet +}$	$=NH^+$	N^+/N ratio[1]
ex-situ 12nm	0.43	0.35	0.22	0.57
ex-situ 22nm	0.56	0.28	0.16	0.44
ex-situ 12nm	0.77	0.18	0.06	0.23
ex-situ 22nm	0.86	0.10	0.05	0.14

[1]Values were calculated using the N(1s) core spectra of the samples.

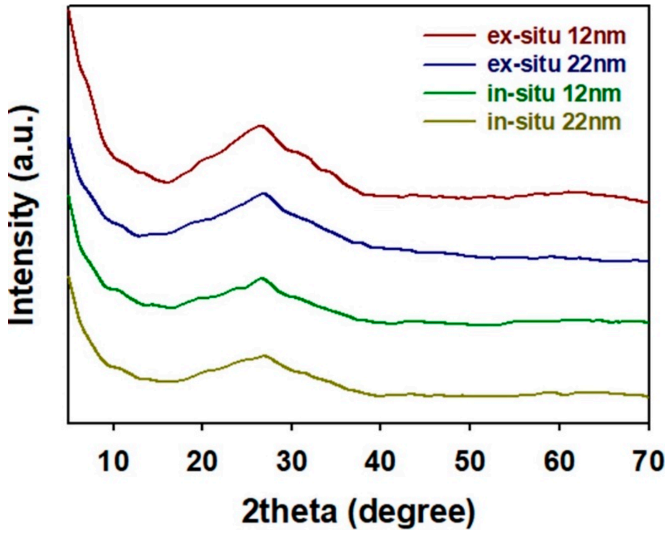

Figure S2. XRD patterns of ex-situ 12nm (red), ex-situ 22nm (blue), in-situ 12nm (green), and in-situ 22nm (olive green).

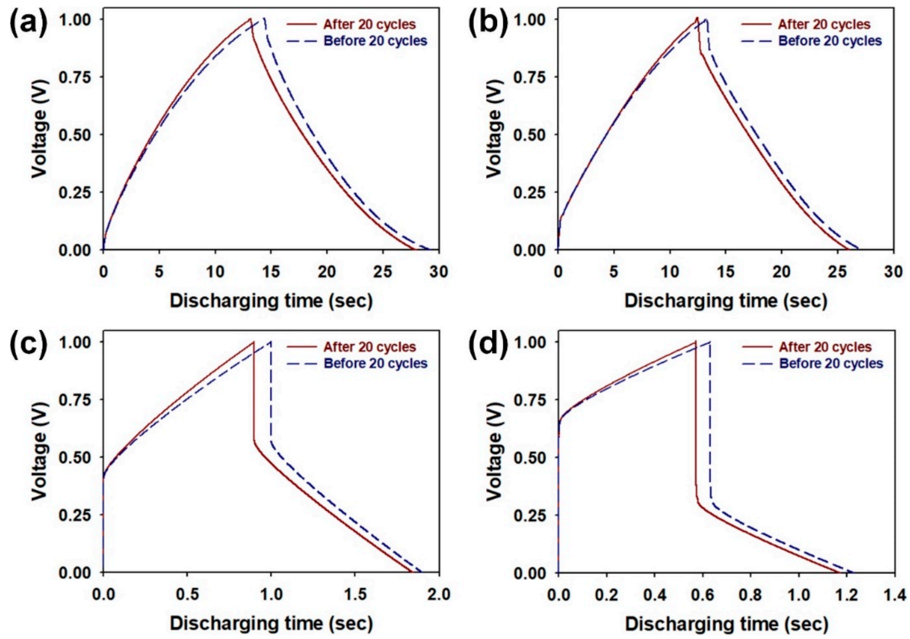

Figure S3. GCD curves of coin cells containing core-shell NPs before and after performing 20 CV cycles at a current of 1 mA/cm^2: (**a**) ex-situ 12nm; (**b**) ex-situ 22nm; (**c**) in-situ 12nm, and (**d**) in-situ 22nm.
